# Cardiac Autonomic Activity, Personality Traits, and Academic Performance in First-Year Medical Students: A Gender-Specific Relation

**DOI:** 10.7759/cureus.49087

**Published:** 2023-11-20

**Authors:** Elizabeth Tharion, Upasana Kachroo, Joseph Noel, Prasanna Samuel

**Affiliations:** 1 Department of Physiology, Christian Medical College, Vellore, Vellore, IND; 2 Department of Physiology, All India Institute of Medical Sciences, Raipur, Raipur, IND; 3 Department of Psychiatry, Christian Medical College, Vellore, Vellore, IND; 4 Department of Biostatistics, Christian Medical College, Vellore, Vellore, IND

**Keywords:** educational psychology, youth, adolescents, personality traits, first-year medical students, heart rate variability, five-factor personality, cardiac autonomic function, students, academic performance

## Abstract

Background

It is not always the sincere or hardworking or intelligent student that gets the highest grades. Exploring unknown dimensions that may distinguish academic performance in adolescents/youth migrating from a high school study environment to that of a professional school and in a learning environment without parental supervision for the first time remains important. We hypothesized that cardiac autonomic activity influenced by cognitive domain factors and emotions would predict academic success in them. Further, we investigated which of their personality traits related to academic performance. Exploratory gender-based analysis was included.

Methods

A prospective cohort study measured first-year medical students' resting heart rate, heart rate variability (HRV), and personality traits (from the self-reported NEO Five-Factor Inventory-3). Spearman's correlation coefficient tested the correlation between the year-end final aggregate marks and assessed parameters, including subgroup analysis based on gender. Regression analyses of variables with academic marks were performed in the entire cohort.

Results

The aggregate marks of 81 volunteering students (*M*_age _= 18.7, *SD *= 0.8 years; 42 females, 39 males) as a cohort did not correlate with their resting heart rate or HRV indices. Subgroup analysis revealed a positive correlation between marks and high-frequency power (r = 0.33, p= 0.03) and total power (r = 0.37, p= 0.02) of HRV in females. The marks positively correlated with the personality conscientiousness score (r = 0.32, p= 0.04) and extraversion score (r = 0.34, p= 0.03) in females. Multivariable regression analysis in the entire cohort revealed no significant interactions.

Conclusion

Academic performance was significantly related to cardiac autonomic modulation and personality traits of conscientiousness and extraversion in female but not male first-year medical students. These results indicate a gender-specific difference in the relation between scholastic performance and HRV in adolescents/youth transiting from high school to professional schools and entering a study environment without parental supervision for the first time. Further our data expands the knowledge base of educational psychology among them.

## Introduction

Success in academia depends on factors in the cognitive realm, like intelligence [1], and on factors in the emotional sphere, such as personality traits and motivation [2-5]. These factors in turn vary in students, based on their genetic makeup, epigenetic influences, cultural background, and environmental influence. Studies link cardiac autonomic activity to emotions [6] and cognitive processes [7]. Hence, we reasoned that academic achievement in the student population may be related to their cardiac autonomic activity. The relationship between cognition, emotions, and cardiac autonomic activity is explained by the neurovisceral integration model by Thayer et al. [8], which describes the connection between neural structures involved in cognition (the prefrontal cortex), affect (the limbic system), and cardiac autonomic control (the medullary cardiac autonomic centers) and forms the basis of the hypothesis of this study.

The resting heart rate is determined by the ratio of the cardiac sympathetic tone to parasympathetic tone (cardiac sympatho-vagal balance). The cardiac vagal (parasympathetic) tone reduces the firing rate, while the cardiac sympathetic tone increases the firing rate of the pacemaker of the heart [9]. A low heart rate is reflective of low cardiac sympatho-vagal balance and vice versa [10]. Given that stress activates the sympathetic nervous system, while the parasympathetic system dominates in conditions of rest and concord [11], we hypothesized that lower resting heart rates would be associated with calmer demeanors and therefore with higher academic outcomes. Further, evidence suggests that higher resting heart rates are associated with greater cognitive decline, as reported in middle-aged individuals [12] and as concluded by a recent meta-analysis [13]. These reports suggest the relation of resting heart rate to cognition and support our hypothesis.

The spontaneous fluctuations in millisecond duration of the cardiac cycle period of adjacent heartbeats, known as heart rate variability (HRV), reflect the modulations of the cardiac autonomic supply [14]. An analysis of the HRV by standardized mathematical procedures gives indices which quantifies the modulations of cardiac autonomic activity by various internal inputs, such as respiration, baroreceptor afferents, and prefrontal and limbic cortex responses [9,14]. A higher HRV indicates a greater malleability of the cardiac autonomic supply to respond to challenges and is indicative of good health [9]. We hypothesized that the HRV of a student incorporate the influence of the cognitive and emotional domain factors on the cardiac autonomic nervous system and, therefore, can predict academic achievement. Studies consistently report the relation of higher cognitive performance to higher HRV [7,15,16], which support our hypothesis. Thus, test scores for global cognitive performance, processing speed, and working memory were found to positively relate to time-domain HRV indices [15]; attention-switching and inhibitory control tests of executive function were found to be related to cardiac vagal control (high-frequency (HF) power of HRV) [7]; and higher cognitive performance in an episodic verbal memory task was found to be associated with higher time-domain HRV indices [16].

The neurovisceral integration model [8] justifies our hypothesis as it provides evidence for the association of HRV with cognitive performance and affect. This model proposes that HRV is an index of the central nervous system functioning that promotes goal-directed behavior and is a marker of health. Our hypothesis is further justified by publications that provide evidence for the relationship of HRV to the cognitive function of the prefrontal cortex and emotional input from the limbic system [17,18]. This published literature lends further support that HRV being related to cognition, affect, and motivation could be an objective predictor of academic performance in first-year medical students. Published literature reveal that HRV remains related to cognitive function even after controlling for other factors such as demography, BMI, presence of diseases affecting cardiac autonomic function, and behaviors such as smoking and exercising [7] and independent of age, race, ethnicity, sex, and education [15,16]. Hence, we hypothesized that irrespective of the factors that might influence HRV, academic performance may be related to HRV. In this exploratory study, we consider HRV indices to be surrogate markers of factors that enhance academic success and thus provide an objective indicator of first-year medical students who might perform poorly in the educational setting. Early identification of such students would be useful to implement timely scholastic interventions which would benefit the students and open more opportunities for growth and development.

We also examined which personality traits of the first-year medical students would influence their academic performance, considering that they form a distinct group of student population who are in their late adolescence/youth, moving from a high school environment to that of a professional school, and in the Indian context entering a learning atmosphere without parental supervision for the first time. Is the academic success of first-year medical students related to their cardiac autonomic functioning? Academic performance is related to which personality traits among them? We addressed these questions and tested our hypothesis by studying the correlation of the year-end final aggregate marks of first-year medical students with their (a) resting heart rate, (b) short-term HRV indices, and (c) measures of the different personality traits. The relationships were also studied based on gender.

## Materials and methods

Study design, setting, and participants

We conducted a prospective cohort study on the first-year medical students of Christian Medical College, Vellore, Tamil Nadu, India, whose annual intake was 100. With the undergraduate medical training program of the institution being a residential course, all students are required to stay in the campus accommodation provided. The study was conducted from January 2017 to March 2018. Students who responded to flyers requesting volunteers to participate in the study were enrolled after obtaining written informed consent. Exclusion criteria included an age of less than 18 years. The institutional review board and ethics committee approved the study (approval number: IRB Min. No. 9931).

Sample size

As the study was exploratory in nature with the study group being a pool of newly joined students, a non-probability sampling technique was utilized. This meant that we performed convenience sampling of all first-year medical students who volunteered for the study and satisfied the eligibility criteria.

Data measurements

All data, except the year-end aggregate academic marks, were collected at a single-point contact with the students. Anthropometric data were measured to calculate the BMI. Sociodemographic details like age, gender, birth order, place of prior schooling (rural/urban), years of education (prior to joining medical school), medical comorbidity, and substance use were collected in a specially designed proforma. After 20 minutes of supine rest in a quiet room, a five-minute digital recording of lead II electrocardiogram (ECG) and respiratory chest movements using a data acquisition system (MP150, BIOPAC Systems, Inc., Goleta, California, United States) was done and stored in computer for subsequent HRV analysis. All recordings were done between 3:00 PM and 7:00 PM, at least two hours after the last meal, and in the proliferative phase of the menstrual cycle in female subjects. Participants had been instructed to abstain from caffeinated beverages/tobacco/alcohol for 12 hours and strenuous activity for 24 hours prior. The supine blood pressure was recorded by a manual sphygmomanometer. Subsequently, standardized questionnaire was administered to assess the personality traits.

Resting supine heart rate

The average heart rate was computed from the five-minute ECG recorded in the supine position, after supine rest of 20 minutes. We have found that the cardiovascular adjustments following the change in position from standing to supine mostly stabilize by 20 minutes of supine rest; hence, we have standardized this protocol in our laboratory.

Short-term HRV indices

Following the guidelines of the Task Force of the European Society of Cardiology and the North American Society of Pacing and Electrophysiology [14], frequency-domain indices of short-term HRV were computed by non-parametric spectral analysis of the RR interval time series of the five-minute recorded ECG, using a specialized software (Nevrokard aHRV version 12.0.0, Medistar Inc., Ljubljana, Slovenia). Briefly, the frequency-domain parameters computed by fast Fourier transformation were the powers in the low-frequency (LF) range of 0.04-0.15 Hz and in the HF range of 0.15-0.47 Hz and the total power (sum of LF and HF powers). The LF power is considered to represent the modulations due to cardiac sympathetic activity, although parasympathetic contribution cannot be discounted [14]. The HF power is widely accepted as the surrogate of cardiac parasympathetic fluctuations, while the total power (LF + HF powers) mirrors the total variability due to modulations of both arms of the cardiac autonomic innervation.

Personality traits

The self-report version of the NEO Five-Factor Inventory-3 (NEO-FFI-3) [19] was administered to the students to assess the prominent personality traits in the five major domains of openness, conscientiousness, extraversion, agreeableness, and neuroticism. The questionnaire is a 60-item inventory with a 5-point Likert response format for each item that takes 10-15 minutes to complete. The completed questionnaires were analyzed, and validated reports were generated with scores in each of the five personality traits. The NEO inventory was chosen as it is reported to be reliable and valid across various cultures [20] and suited for non-native English speakers, including those from India [21]. We specifically selected NEO-FFI-3 as it is a shortened version and best suited the needs of the study for a quick and accurate assessment of the personality traits of the volunteering students.

Academic performance

The students were followed up till the end of the first year of the medical course to obtain their year-end aggregate academic marks. Towards this, the composite marks of the three subjects (Anatomy, Biochemistry, and Physiology) of the first-year course were computed, including the internal assessment marks and the marks obtained in the year-end examinations held by the University, a state body. The final aggregate marks thus calculated at the end of the first year of medical school were considered as an index of academic performance and correlated with the resting heart rate, the HRV indices, and the score obtained in each of the five personality traits.

Statistical analysis

All participants were assigned an identification (ID) number to maintain anonymity. Data were entered into the EpiData software (EpiData Association, Odense, Denmark) and statistically analyzed with the Stata Statistical Software: Release 11 (2009; StataCorp LLC, College Station, Texas, United States). Normally distributed data are described as mean (*M*) and standard deviation (*SD*), while non-normally distributed data are described as median with inter-quartile (*Q1-Q3*) values. As the HRV indices were non-normally distributed, Spearman's rank-order correlation test was applied to study the correlations between parameters in the entire cohort and in the gender-based subgroup analysis. Bivariate linear regression analyses were done to quantify the effect of different covariates on academic performance (outcome variable) in the entire cohort. All variables that were found to be significant at p < 0.10 were included in the multivariable analyses, adjusted for gender, to obtain adjusted estimates. Beta coefficients with 95% confidence interval (CI) were reported from linear regression analyses. A p-value of < 0.05 was considered for statistical significance. 

## Results

Of the 100 students in the first year of the medical course, 81 students (*M*_age_ = 18.7, *SD* = 0.8 years; 42 females, 39 males) volunteered and were found eligible to take part in the study. The demographic data and baseline variables of study participants are given in Table 1.

**Table 1:** Demographic and baseline variables of participants (overall and by gender) bpm: beats per minute; BMI, body mass index; LF, low frequency; HF, high frequency

Variables	Mean (SD)/median (Q1-Q3)/percentage		
	Overall sample	Males	Females
Sample, no.	81	39	42
Age (years)	18.65 (0.82)	18.51 (0.72)	18.79 (0.90)
Birth order			
First	59.3%	58.97%	59.52%
Second	34.6%	30.77%	38.10%
Third	6.17%	10.26%	2.38%
Place of prior schooling			
Urban	88.89%	89.74%	88.10%
Rural	11.11%	10.26%	11.90%
Years of prior education (y)	12.51 (0.64)	12.49 (0.68)	12.55 (0.59)
Medical comorbidity			
No	95.06%	94.87%	95.24%
Yes	4.94%	5.13%	4.76%
Substance use			
No	98.77%	100.00%	97.62%
Yes	1.23%	0%	2.38%
BMI (kg/m^2^)	22.58 (3.68)	21.91 (3.77)	23.21 (3.53)
Respiratory rate (cycles/min)	18 (4)	17 (5)	19 (3)
Heart rate (bpm)	70 (8)	68 (7)	72 (9)
Mean arterial pressure (mmHg)	79 (5)	82 (5)	77 (5)
LF power (ms^2^)	870.27 (417.56-1654.19)	949.56 (501.92-2203.58)	800.30 (383.33-1604.47)
HF power (ms^2^)	1348.49 (636.84-2160.26)	1037.48 (589.26-1697.77)	1612.29 (757.75-3482.86)
Total power (ms^2^)	2262.87 (1321.2-4661.19)	1850.41 (1321.20-4295.92)	2635.49 (1231.35-4958.81)
Neuroticism score	23.91 (8.23)	23.77 (7.43)	24.05 (8.99)
Extraversion score	29.33 (6.81)	29.46 (6.40)	29.21 (7.24)
Openness score	32.26 (6.27)	31.77 (5.40)	32.71 (7.01)
Agreeableness score	30.47 (6.08)	29.67 (5.81)	31.21 (6.30)
Conscientiousness score	27.90 (7.64)	25.33 (6.29)	30.29 (8.06)
Aggregate marks (out of 600)	391.49 (54.86)	371.18 (45.98)	410.36 (56.18)

Academic marks did not correlate with the resting heart rate nor with the HRV indices, when considering the entire cohort of students (Table 2). However, subgroup analysis based on gender revealed that the academic marks correlated positively with the HRV indices in female students, but not male students (Table 2). In females, the grades correlated positively with the HF power (r = 0.33, p = 0.03) and the total power of HRV (r = 0.37, p = 0.02).

**Table 2:** Correlation of aggregate marks with resting heart rate, HRV indices, and personality trait scores (overall and gender-based) HRV: heart rate variability; LF, low frequency; HF, high frequency ^†^Spearman's rho correlation coefficient *Statistically significant p-values (p < 0.05)

Variables	Correlation of marks					
	Overall sample (n = 81)		Males (n = 39)		Females (n = 42)	
	rho^†^	p-value	rho^†^	p-value	rho^†^	p-value
Resting heart rate	-0.03	0.80	-0.09	0.61	-0.16	0.32
LF power	0.09	0.44	-0.01	0.95	0.29	0.07
HF power	0.09	0.41	-0.30	0.60	0.33	0.03*
Total power	0.13	0.25	-0.16	0.33	0.37	0.02*
Neuroticism score	-0.07	0.54	-0.01	1.00	-0.13	0.40
Extraversion score	0.19	0.09	0.02	0.90	0.34	0.03*
Openness score	0.16	0.16	-0.14	0.40	0.30	0.05
Agreeableness score	0.07	0.54	-0.08	0.65	0.10	0.55
Conscientiousness score	0.38	0.00*	0.26	0.11	0.32	0.04*

The correlation analysis between academic performance and personality traits revealed that marks achieved correlated positively with the conscientiousness score (r = 0.38, p < 0.05; Table 2), for the entire group of students. Gender-based analysis reflected the same finding in female but not male students (Table 2). Academic scores of females correlated positively with their conscientiousness score (r = 0.32, p = 0.04). In addition, the marks of females correlated positively with their extraversion personality score (r = 0.34, p = 0.03).

The results of the bivariate linear regression analyses showed only extraversion score and the conscientiousness score to be significant at p < 0.10 (Table 3). Hence, they were included in the multivariable analysis for the entire cohort, adjusted for gender. The results showed that there was no significant interaction between gender and conscientiousness score or extraversion score (Table 3).

**Table 3:** Regression analyses of variables with academic marks in the entire cohort (n = 81) LF, low frequency; HF, high frequency *Variables with p-values < 0.10 in bivariate analyses were included in the multivariable analyses, adjusted for gender

Variables	Bivariate analyses		Multivariable analyses*	
	(Unadjusted) beta coefficient (95% CI)	p-value	(Adjusted) beta coefficient (95% CI)	p-value
Gender	-39.18 (-61.98, -16.37)	0.001	-30.32 (-53.57, -7.08)	0.011
Resting heart rate	-0.19 (-1.72, 1.33)	0.800		
LF power	0.00 (-0.01, 0.01)	0.864		
HF power	0.00 (0.00, 0.01)	0.549		
Total power	0.00 (0.00, 0.00)	0.629		
Neuroticism score	-0.47 (-1.96, 1.02)	0.535		
Extraversion score	1.54 (-0.23, 3.31)	0.088	1.05 (-0.62, 2.73)	0.215
Openness score	1.37 (-0.57, 3.31)	0.163		
Agreeableness score	0.63 (-1.39, 2.64)	0.537		
Conscientiousness score	2.73 (1.24, 4.22)	0.000	1.83 (0.25, 3.42)	0.023

## Discussion

The objectives of our study were to investigate the relationship between first-year medical students' academic performance and their cardiac autonomic activity and to explore which of their personality traits related to academic outcomes. We had expected students with lower resting heart rates and higher total HRV to achieve greater academic success. We found that the academic achievement of the first-year medical students of our study had no relation to their resting heart rate but had a gender-based relation with their HRV indices. Female students with higher HF power (that reflect greater modulation of the cardiac parasympathetic activity), as well as higher total power of HRV (that reflect greater overall variability of both arms of the cardiac autonomic supply considered together), scored greater aggregate marks. The male students did not exhibit any relation between their cardiac autonomic metrics and academic achievement.

Further, we observed that students with a higher conscientiousness score in the personality trait performed better academically. This relation between conscientiousness score and academic marks was seen in females but not in males, when examined separately. Besides, females with higher extraversion scores achieved more marks. We did not observe any relation between the personality scores and academic success in the males of our study population of first-year medical students.

Given that several studies report a robust relationship between HRV and cognition despite presence of confounders that may affect HRV, such as demographic variables, BMI, diseases affecting cardiac autonomic activity, smoking status, and physical activity level [7,15,16], we had expected that academic performance could be correlated with HRV indices in our student population. While no significant results were found when considering the entire cohort of student population, notably gender-based analysis revealed a compliance with our hypothesis in female students, but not in male students. We found that the females who gained higher grades also had higher HRV. HRV denotes the extent of modulation of the cardiac sympathetic and parasympathetic supply and is a measure of the responsiveness of the cardiac autonomic supply [22]. This implies that females with greater cardiac autonomic responsiveness scored more marks. Considering our data in the light of the neurovisceral integration theory [8,17,18], we suggest that factors which enhanced cardiac autonomic modulation in adolescent females may also have positively impacted academic achievement in them. We did not find a similar relationship between HRV and academic success in adolescent males.

Several studies report the relationship between resting heart rate and other conditions and variables. Thus, it has been observed that low heart rate in children predicts adverse outcomes in social domains of their development [23]. A high resting heart rate has been reported in anxiety disorders [24]. On the other hand, men with higher intelligence quotient (IQ) were found to have a significantly lower heart rate (higher mean R-R interval) than men with average IQ, in a study conducted on post-myocardial infarction patients [25]. We had postulated that the academic success of first-year medical students would vary inversely with their resting heart rate, as they transit through late adolescence and youth. Contrary to our assumptions, we found no relation between resting heart rate and academic performance. This finding does not support studies that report greater cognitive decline in those with higher resting heart rates [12,13].

Similarly, published reports of prior investigations detail the relationship between HRV and various other factors and circumstances. Thus, low HRV has been noted in anxiety disorders [24], while high HRV has been documented in men with higher IQ [25]. We had hypothesized that the academic merit of the first-year medical students of the current study would vary directly with their total HRV. We found that the final aggregate marks significantly varied positively with the HRV of the female students, a relationship which we did not observe in the male students nor when considering the entire cohort of the first-year medical students, revealing a gender-dependent relation between academic performance and cardiac autonomic activity in our study population of adolescents/youth. The current study explored if estimation of HRV would be able to distinguish students who may perform poorly in academics. Our analysis revealed that a single assessment of HRV could provide the necessary information, although in female students only and in our distinct study population of first-year medical students. The findings of our study need to be followed up with further investigations in varied student populations.

The gender variation in the relation between academic achievement and personality traits that we report is an interesting observation in the context of factors impacting academic success during the period of late adolescence. While females with higher conscientiousness and extraversion scores were more academically successful, the academic scores of males did not vary with any of their personality trait scores. Conscientiousness and extraversion are personality traits that have been reported to be positively correlated with better academic performance in students [26]. Extraversion is a trait that leads a person to go out and mingle with others and was found to be strongly linked to academic outcome in the context of the flipped classroom teaching [27]. However, higher trait scores in extraversion have been reported to also lead to greater engagement in social activities and attention-drawing behaviors at the expense of other important tasks [28]. Based on this line of thinking, it may be argued that males with higher extraversion scores may spend more time socializing instead of studying and therefore their academic marks do not correlate with the trait scores. On the contrary, adolescent females with higher extraversion scores may associate more with others academically and thus achieve higher scores, as has been reported in the setting of the flipped classroom teaching [27]. These arguments put forth gender-specific consequences of a given personality trait during the period of late adolescence. They also indicate a gender difference among the adolescent/youth group in their approach to academic activities with females advancing their knowledge collaborating with others, while males fail to take advantage of the academic benefits of interacting with others. Similarly, though conscientiousness is a personality trait marked by diligence and persistence [29], higher conscientiousness score did not translate to higher academic marks in our study population of adolescent males. Published literature suggests that male students differ from females in their awareness of self-regulated learning and knowledge of study strategies [30].

The statistically significant correlations that we report in this study, though apparently weak because of low values of Spearman's rho, are nevertheless noteworthy, as they measure the relationship between variables which are difficult to measure. In addition, these measurements were done in first-year medical students who form a distinct group among student populations for several reasons. They were in the age group of late adolescence/youth transiting to adulthood, moving from the setting of high school teaching-learning to that of professional school and exposed to an environment of learning without parental supervision for the first time. Our findings contribute to the understanding of the gender difference in educational psychology among this distinctive group of adolescent/youth student population. Importantly, the correlation we report between academic performance and HRV in the female students of this population is noteworthy and in line with previous literature that associate HRV to cognition and affect [7,8,15-18].

A constraint of our study is the homogeneity of the study population chosen and therefore the limits to generalizability of results. Medical students are a biased sample of the student population, of highly motivated individuals with low variance in academic achievement. Despite this limitation, we could expose a gender-specific relationship between HRV and academic grades in the medical student population of our study. Additional investigations in appropriate student populations will further establish and unravel the nuances in the relationship between academic grades and objective markers of cardiac autonomic functioning. Another limitation of our study is that we did not consider the influence of factors not captured by HRV which may have had a bearing on academic performance.

## Conclusions

A central objective of our study was to examine cardiac autonomic function as antecedents of academic achievement in first-year medical students. Our findings demonstrate that the relationship between academic success and HRV is influenced by the gender of the students: significant positive coefficients between aggregate marks and HRV indices being observed in females, but not males. We also observed a gender-dependent relation between academic performance and personality traits: while females with higher personality conscientiousness and extraversion scores were more academically successful, the academic merit of males did not associate with any of their personality traits. Our data contribute to understanding the educational psychology and the relationship between academic performance and HRV among adolescents/youth, who are migrating from the high school study environment to that of a professional school and entering a learning environment without parental supervision for the first time. In the larger context, the purpose of this study was to find out if estimation of the HRV indices would help in the early identification of students who may fare poorly in the educational setting. Our exploratory study revealed that a single assessment of HRV could provide the necessary information in our study population, albeit in female students only. Communicating the outcomes of our study will help researchers follow up our findings with further investigations in diverse student populations.
